# Learning-Based Near-Infrared Band Simulation with Applications on Large-Scale Landcover Classification

**DOI:** 10.3390/s23094179

**Published:** 2023-04-22

**Authors:** Xiangtian Yuan, Jiaojiao Tian, Peter Reinartz

**Affiliations:** German Aerospace Center (DLR), Münchner Str. 20, 82234 Weßling, Germany; xiangtian.yuan@dlr.de (X.Y.); peter.reinartz@dlr.de (P.R.)

**Keywords:** multispectral, remote sensing, NIR, RGB, cGAN, Sentinel-2, SEN12MS, robust loss, SSIM

## Abstract

Multispectral sensors are important instruments for Earth observation. In remote sensing applications, the near-infrared (NIR) band, together with the visible spectrum (RGB), provide abundant information about ground objects. However, the NIR band is typically not available on low-cost camera systems, which presents challenges for the vegetation extraction. To this end, this paper presents a conditional generative adversarial network (cGAN) method to simulate the NIR band from RGB bands of Sentinel-2 multispectral data. We adapt a robust loss function and a structural similarity index loss (SSIM) in addition to the GAN loss to improve the model performance. With 45,529 multi-seasonal test images across the globe, the simulated NIR band had a mean absolute error of 0.02378 and an SSIM of 89.98%. A rule-based landcover classification using the simulated normalized difference vegetation index (*NDVI*) achieved a Jaccard score of 89.50%. The evaluation metrics demonstrated the versatility of the learning-based paradigm in remote sensing applications. Our simulation approach is flexible and can be easily adapted to other spectral bands.

## 1. Introduction

Multispectral remote sensing is one of the important means of Earth observation. It has been extensively employed to explore the physical, chemical and biological properties of the Earth’s surface. In addition to visible spectra that the human visual system can perceive, multispectral sensors capture signals from additional spectral ranges. Since ground objects respond differently to light of certain wavelengths, the wider spectral range allows additional information to be extracted from ground objects.

Due to limitations of budget, technology, intended application and various other reasons, not every sensor is capable of capturing a wide range of wavelengths across the electromagnetic spectrum. Moreover, differences in the wavelength characteristics of different sensors can make it challenging to use data from multiple sensors simultaneously, necessitating the process of harmonization [1]. Some pixels might be corrupted during the data down-link from satellites, which can hinder further analysis [2]. In the case of unmanned aerial vehicle (UAV) remote sensing, low-cost, off-the-shelf cameras typically capture only visible light in the red, green, and blue wavelengths, which limits their potential for downstream applications that require near-infrared spectra, such as vegetation monitoring. As a result, researchers have modified commercial cameras to capture the *NIR* bands, but registration of each band is often required [3,4,5,6].

Among the common spectral bands, the near-infrared (*NIR*) bands have been used extensively in Earth observation tasks. In combination with visible spectra, *NIR* bands contain additional features of ground objects, especially vegetation. For example, indices using *NIR* bands have been developed for tasks such as land cover classification. These indices include the normalized difference vegetation index (*NDVI*) and the normalized difference water index (*NDWI*), which have been shown to be effective in highlighting vegetation and open water in remotely sensed imagery [7]. In addition to identifying vegetation and water, *NIR* spectroscopy can also help detect materials such as plastics [8,9], certain minerals [10], and tree health problems [11,12]. In addition, *NIR*-derived indices have also been used in tasks such as atmospheric correction [13]. In data-hungry machine learning or deep learning methods for land cover classification, *NIR* bands are able to improve coarse ground truth or correct mislabelling for some classes that are sometimes challenging for the human eye to interpret. Therefore, the generation of additional spectral bands from known bands has potential practical applications in Earth observation but has not yet been extensively explored. However, the underlying problem is that there are no precise physical models that map a spectral response from another wavelength. Signals that the sensor receives depend on many factors, including atmospheric effects, weather condition, land cover type, and terrain, etc. Ignoring these effects, we want to test whether a simple end-to-end model is sufficient to generate additional bands from known bands on a large scale, without the myriad of input parameters of complicated models.

The generation of artificial *NIR* bands using only the visible spectrum can be considered as a nonlinear mapping problem. Neural networks have been shown to be effective in nonlinear mapping [14]. It could also be viewed as a generative problem, which can be addressed by neural networks and especially GANs. Unlike computer vision tasks, which usually have some level of abstraction from the input, our task is to ensure that the generated NIR band is also consistent in structure and spatial distribution. To this end, additional loss functions, such as L1 or L2, are added to the GAN loss to ensure that the output is close to the ground truth [15]. However, such losses are prone to outliers. Several robust loss functions are able to handle outliers by being less sensitive to large errors. A single robust loss function proposed by Barron [16] integrates several common robust loss functions that are controlled by a single continuous-valued parameter that can also be tuned when training neural networks.

In our preliminary work, we performed experiments on a dataset acquired in summer [17]. It was shown that a conditional GAN model was able to generate a realistic *NIR* band from the visible spectrum. The generated *NIR* band retained the original image’s texture and correctly displayed the spectral characteristics of certain land cover. However, in this proof of concept work, only a relative small dataset was used for the evaluation to eliminate the potential influence of the seasons on the results. In this work, we will present more details about the method to generate artificial spectral bands from the visible bands using cGAN. The method is tested with the complete SEN12MS dataset with four full seasons. In addition to the pixel-wise evaluation, by comparing with the ground truth, a rule-based land cover classification is carried out to assess the quality of the artificially generated *NIR* band in a downstream remote sensing application.

The contribution of our work is twofold: based on our previous proof of concept work, we integrated a structural similarity loss to further improve the performance of the method; we evaluated the method with the complete four seasons of the SEN12MS dataset, validating the effectiveness and revealing the limitations of this method.

The paper is structured as follows: In Section 2, we review relevant literature; in Section 3, we introduce the model in detail and the dataset used in this study; in Section 4, we describe the experimental settings; in Section 5, the results are presented and analyzed, followed by the conclusion in Section 6.

## 2. Related Work

Traditional techniques for solving remote sensing problems have been continuously challenged by machine learning methods over the last decades. Machine learning methods have been extended to remote sensing images, which have some peculiarities compared to natural images. Among machine learning methods, GANs have gained increasing attention due to their versatility and performance. For example, GANs have been applied to tasks such as image super-resolution and pan-sharpening. Sajjadi et al. [18] proposed a GAN-based network to super-resolve natural images using automated texture synthesis combined with a perceptual loss that produced realistic texture, instead of just approximating the ground truth pixel during training. Jiang et al. [19] applied a GAN-based model with an edge enhancement subnetwork for super-resolution, which effectively removed noise in the reconstructed image and enhanced the contour of ground objects. This model takes advantage of the adversarial learning strategy, which is insensitive to noise. GAN-based models have also been applied in remote sensing image pre-processing, such as image dehazing and cloud removal. Enomoto et al. [20] adopted NIR bands with an RGB image in a GAN-based framework to remove clouds from satellite images. Grohnfeldt et al. [21] fused Sentinel-1 SAR data and Sentinel-2 optical multi-spectral imagery in a GAN framework to produce cloud-free optical images, which has shown more robust performance due to the properties of the synthetic aperture radar (SAR) sensor. GAN-based models have been applied to treat many problems as generative, such as monocular height estimation [22,23], DSM refinement [24], PAN-sharpening [25], image super-resolution [26], and change detection [27].

One way of approaching the spectral band simulation problem is to treat it as a classification problem. Within this framework, the mapping process could be viewed as involving the injection of spectral signatures into corresponding classes of ground objects. In recent years, neural networks have been widely used for land cover classification and have become a popular classification paradigm in the remote sensing community. The problem could also be viewed as a generative one as well, and, thus, can be tackled by neural-network- and GAN-based methods. Therefore, it is theoretically possible to simulate spectral reflectance from other bands using neural-network-based methods. Specifically, hyperspectral or multispectral image reconstruction, in which responses at multiple electromagnetic wavelengths need to be simulated, is a hot research top in spectral simulation using deep learning methods. The paper by Fu et al. [14] proposed a network structure for hyperspectral image reconstruction from RGB bands. The network consists of a spectral sub-network, which performs the spectral nonlinear mapping, and a spatial sub-network, which models the spatial correlation. The network uses a traditional loss function (mean square error) to force the generated bands to be numerically similar to the real ones. Deng et al. [28] proposed a neural-network-based method (M2H-Net) to reconstruct hyperspectral image from arbitrary number of input bands within spectral range of 300 to 2500 nm. The method was verified by UAV and satellite data captured at different locations in China. Zhao et al. [29] used a model trained by a hyperspectral benchmark dataset WHU-Hi-Honghu HSI [30] to convert true RGB to natural RGB, which was subsequently used with its multispectral pair to train an HSCNN-R network [31] for reconstruction. The model was trained with a multi-temporal and multispectral dataset of a maize field and successfully tested on the imagery of a rice field. Like many other remote sensing issues, the hyperspectral reconstruction work can be tackled by GAN-based methods as well. Alvarez-Gila et al. [32] used a conditional GAN to reconstruct a hyperspectral image from an RGB image. The method was trained and tested on 201 natural images of 1392×1300. Liu and Zhao [33] proposed a scale attention pyramid UNet (SAPUNet) that adopted a dilated convolution for feature extraction and an attention mechanism for feature selection. SAPW-Net was proposed in the same work, with an additional branch for boundary supervision. The work achieved improved results on the interdisciplinary Computational Vision Lab at Ben Gurion University (ICVL) dataset [34].

Due to the prohibitively high cost of hyperspectral imagery, the amount of open-source hyperspectral datasets is limited, and the size of the available datasets is relatively limited. According to the review by [35], the available open-source hyperspectral datasets are only of small to medium size [35]. However, multispectral datasets have better availability, and many satellite missions have global coverage, such as the Sentinel mission, enabling large-scale experiments and analysis.

The integration of additional NIR bands in cameras has practical applications in various remote sensing research fields, including vegetation monitoring. However, the widespread adoption of NIR-capable cameras is limited by cost and technical constraints [3]. To overcome this, numerous researchers have modified commercial RGB cameras to enable the capture of additional near-infrared band radiation for vegetation and soil monitoring by unmanned aerial vehicles (UAVs). For instance, Rabatel et al. [3] removed the NIR blocking filter and added an external long-wavelength pass filter to a single commercial camera (Canon 500D). The optimal external filter was determined by BSOP (band simulation by orthogonal projection), which relies on known sensitivity curves of the camera. Other studies employed two cameras to capture RGB and NIR images separately [4,5], which requires accurate pixel alignment. Brown and Süsstrunk [6] created a 477-image RGB-NIR dataset captured by a modified single-lens reflex (SLR) camera. The NIR and RGB bands were registered using a feature-based alignment algorithm [36] with robust estimation of a similarity motion model. The joint entropy analysis suggested that NIR contains significantly different information from the visible bands.

Different from the methods that involve hardware modification, learning-based methods focusing on the image data using generative methods have also been studied. Our previous work [17] was one of the first to apply GANs to simulate NIR bands from RGB bands. Subsequently, several studies have been published to explore the potential of simulated NIR bands. Koshelev et al. [37] synthesized an NIR band to boost the performance of hogweed crops segmentation. Sa et al. [38] proposed a fruit detection dataset with RGB and NIR bands. Aslahishahri et al. [39] curated a dataset with blue, green, red, NIR, and red edge bands covering canola, lentil, dry bean, and wheat breeding fields. These works provide valuable datasets that can be used for specific remote sensing tasks.

## 3. Method and Data

**Figure 1:**
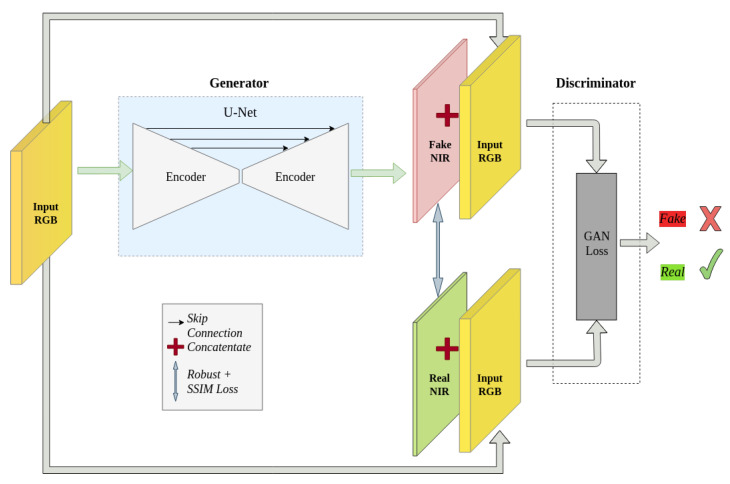
The workflow of the method. The GAN loss is also used for back propagation in the generator, which is not shown in the visualization. The weights of the generator and the discriminator are updated alternately.

In this section, details of our method will be introduced in detail. An overview of the method is shown in Figure 1.

### 3.1. Conditional GAN

A GAN consists of two components: a generator and a discriminator. The generator’s aim is to produce fake results, while the discriminator tries to differentiate between the generator’s output and the real distribution [40]. These two components work in a competitive manner inspired by game theory, where any improvement in one leads to a corresponding deterioration in the other. In a typical setup for vision tasks, GANs take in random noise vectors from a particular distribution and produce realistic-looking images as outputs. In contrast to traditional GANs, conditional GANs (cGANs) [41] require specific input data. In the context of spectral band simulation, the network must generate the *NIR* band that is related to the RGB bands. In addition, the discriminator is also conditioned on the input RGB bands. By attaining equilibrium between the generator and the discriminator, the cGAN can produce a realistic-looking NIR band that corresponds to the input RGB bands.

#### 3.1.1. Generator

The task of generating a realistic NIR band from RGB bands can be considered as a mapping from the visible to the near-infrared spectrum. As the input and output represent the same ground objects, they should possess matching structures, similar textures, and encode the same semantics. Many GAN generators use encoder-decoder structures that downsample the input and then gradually upsample it to the output, which leads to the loss of low-level information from earlier stages and consequently degraded details. Hence, several CNN models use long skip connections to retain fine-grained information from earlier layers that are lost during down-sampling, and, thereby, perform better for tasks that require finer spatial information. Therefore, the encoder-decoder network with a skip connection, famously known as the U-Net structure [42], is well-suited for this task. The U-Net structure comprises eight blocks in both the encoder and decoder, with each block containing convolution, batch normalization, and a leaky rectified linear unit (LeakyReLU) with a slope of 0.2 in the encoder. The decoder blocks comprise transposed convolution, batch normalization, and ReLU layers. The convolution has a filter size of 4 and a stride of 2 in both the encoder and decoder. In contrast to some conditional GANs that use Gaussian noise as input to the generator to avoid deterministic results matching the Dirac delta function [15], our task does not require much randomness, so we do not use this approach. Instead, we use dropout in the generator during both training and testing phases, which reduces stochasticity but is suitable for our task. The first three blocks in the decoder have dropout layers with a rate of 50%.

#### 3.1.2. Discriminator

**Figure 2:**
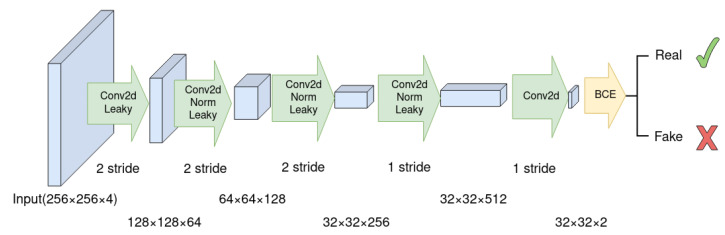
The PatchGAN discriminator, as shown in the diagram, is made up of several blocks comprising of 2D convolutions, a LeakyReLU activation function, and a binary cross-entropy function at the end. The first three convolutions have a filter size of 4×4 and a stride of 2. The last two convolution layers have a stride of 1 to retain the spatial resolution. The output of the discriminator after binary cross-entropy (BCE) is obtained by averaging the outputs of all the patches.

The discriminator should be neither too strong nor too weak with respect to the generator. If the discriminator is too weak, the generator can easily fool it, possibly leading to an unrealistic NIR band. On the other hand, if the discriminator is too powerful, the generator might not pass its scrutiny. There are various options available for the discriminator, depending on the intended purpose. One choice is the Markovian discriminator, also known as PatchGAN [15], as shown in Figure 2. This discriminator classifies whether a patch of size N×N in the input image is real or fake and averages all the patches in the image. It consists of several blocks of 2D convolution, batch normalization, and LeakyReLU layers, with a stride of 2 for all convolutions, except for the last two convolutions. The PatchGAN can be considered a form of texture loss, as it focuses on the local structure of the image.

**Figure 3:**
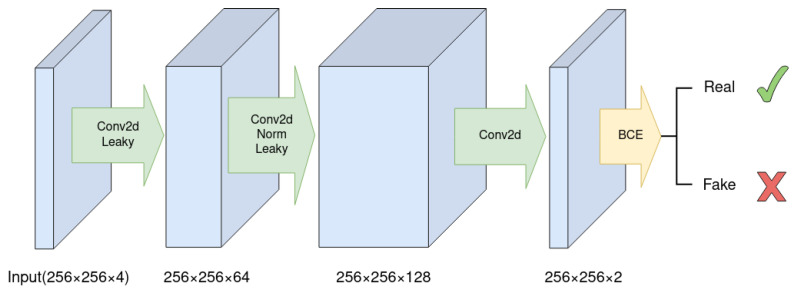
The PixelGAN discriminator. Each 1×1 convolution has a stride of 1. The prediction is only on pixel level. The output is averaged of all the pixels. The PixelGAN discriminator does not encode texture information.

Another option is the pixel discriminator, which operates on the pixel level and does not consider texture information. The kernel size and stride in the pixel discriminator are both 1, keeping the feature map size constant throughout the network. Figure 3 illustrates the architecture of the pixel discriminator. Both discriminators have a binary cross-entropy layer as their last layer, which classifies the generated image as true or false, and the results are averaged over the entire image. When training the cGAN, the weights of the discriminator are updated first, and the weights of the generator are updated subsequently.

### 3.2. Loss Function

The objective in our experiment consists of three terms: conditional GAN loss, robust loss and SSIM loss. The conditional GAN loss is used by both the generator and discriminator for back propagation, while the robust loss and SSIM are only considered by the generators.

#### 3.2.1. GAN Loss

In unconditional GANs, the generator G maps a random noise *z* that follows a certain probability distribution to the output *y*. The discriminator tries to predict the generator’s output as fake. The loss function of the unconditional GANs can be written as:

(1) $$\begin{array}{cc} L_{GAN}\left(G,D\right) & =E_{y}\left[LogD\left(y\right)\right]\\ \; & +E_{z}\left[log\left(1-D\left(G\left(z\right)\right)\right)\right] \end{array}$$

Conditional GANs learn mapping from both random noise *z* and input image *x*: G:{x,z}→y. In our case the noise is realized by dropout. The discriminator *D* is trained adversarially against the generator *G* to distinguish between the real image and the generated image. The objective function of conditional GANs can be expressed as:

(2) $$\begin{array}{cc} L_{cGAN}\left(G,D\right) & =E_{x,y}\left[LogD\left(x,y\right)\right]\\ \; & +E_{x,z}\left[log\left(1-D\left(G\left(x,z\right)\right)\right)\right] \end{array}$$

In detail, the generator tries to maximize log(D(G(x,z))) instead of minimizing the objective $E_{x,z}\left[log\left(1-D\left(G\left(x,z\right)\right)\right)\right]$, as suggested in the original GAN paper [40].

#### 3.2.2. Robust Loss

Combining GAN objectives with traditional losses, such as L1 and L2, has been proven beneficial [43]. In cGAN, the generator should also produce output that is close to the ground truth numerically. Traditional loss functions, such as L1 and L2, are susceptible to noise in the data. They are heavily skewed by outliers that might result from many factors, such as sensor errors, data transmission errors or pre-processing errors. Robustness is a crucial property that is desired in machine learning models. There are several robust loss functions that demonstrate reduced sensitivity to large errors, such as Cauchy/Lorentzian [44], Geman-McClure [45], Welsch [46], Charbonnier [47] and generalized Charbonnier [48]. When the loss is large, some of these loss functions can have a saturated or decreased gradient. To address this issue, a robust loss function has been proposed by Barron [16], which includes several common robust loss functions as a subset. This loss function has the capability to adjust its robustness continuously during training through a parameter α. The general form of the robust loss function can be expressed as:

(3) $$f\left(x,\alpha ,c\right)=\frac{\left|\alpha -2\right|}{\alpha }\left({\left(\frac{{\left(x/c\right)}^{2}}{\left|\alpha -2\right|}+1\right)}^{\alpha /2}-1\right)$$

This loss function is a versatile generalization of several existing loss functions mentioned above. In Equation (3), c>0 represents the scale parameter that determines the size of the quadratic bowl near x=0. Additionally, the robust loss function can be used to construct a general probability distribution, where the negative log-likelihood (NLL) of the probability density is a shifted version of the robust loss function. The distribution is written as:

(4) $$p\left(x|\mu ,\alpha ,c\right)=\frac{1}{cZ(\alpha )}exp\left(-f\left(x-\mu ,\alpha ,c\right)\right)$$

Z(α) in Equation (4) is a partition function defined as:

(5) $$Z\left(\alpha \right)=\int_{-\infty }^{\infty }exp\left(-f\left(x-\mu ,\alpha ,c\right)\right)$$

To simplify the partition function, the logarithmic of Z(α) is approximated using the cubic Hermit spline. The monotonicity of the general loss with respect to α will result in simply minimizing α to reduce the cost of outliers. Therefore, the final robust loss function is the NLL of the general distribution (Equation (4)) instead of the general loss function (Equation (3)). The values for *c* and μ are 1 and 0, respectively. For more details, readers are referred to the paper from Barron [16]. The objective for cGAN with robust loss function can be expressed as:

(6) $$L_{cGAN}\left(G,D\right)+\lambda L_{Robust}\left(G,\alpha \right)$$

(7) $$L_{Robust}\left(G,\alpha \right)=NLL\left(p\left(x|0,\alpha ,1\right)\right)$$

#### 3.2.3. Structural Loss

In addition to the traditional loss and the robust loss, we integrate an additional structural loss function during training. The structural similarity index (SSIM) is a widely used metric for evaluating the perceived quality of generated images [49]. The SSIM index is a comprehensive metric for measuring image similarity within a defined window as it takes into account luminance (l), contrast (c), and structure (s). It is defined as the weighted combination of the three comparative measures:

(8) $$SSIM\left(x,y\right)={\left[l\left(x,y\right)\right]}^{\alpha }\;\cdot \;{\left[c\left(x,y\right)\right]}^{\beta }\;\cdot \;{\left[s\left(x,y\right)\right]}^{\gamma }$$

In Equation (8), the l, c, and s are defined as follow:

(9) $$l\left(x,y\right)=\frac{2\mu_{x}\mu_{y}+C_{1}}{\mu_{x}^{2}+\mu_{y}^{2}+C_{1}}$$

(10) $$c\left(x,y\right)=\frac{2\sigma_{x}\sigma_{y}+C_{2}}{\sigma_{x}^{2}+\sigma_{y}^{2}+C_{2}}$$

(11) $$s\left(x,y\right)=\frac{2\sigma_{xy}+C_{3}}{\sigma_{x}\sigma_{y}+C_{3}}$$

In Equation (8), α,β,γ define the relative importance of the three components. In Equations (9)–(11), $\mu_{x}$ and $\mu_{y}$ are the intensity values of the two windows for comparison; $\sigma_{x}$ and $\sigma_{y}$ are the standard deviations; $C_{1}$, $C_{2}$, and $C_{3}$ are constants to avoid issue caused by zero denominator instability, and are related to the dynamic range of pixel values. A Gaussian sliding window with size of 11 and σ=1.5 is applied to the image. If $C_{3}=C_{2}/2$, and α,β and γ are all equal to 1, Equation (8) simplifies to:

(12) $$SSIM\left(x,y\right)=\frac{\left(2\mu_{x}\mu_{y}+C_{1}\right)\left(2\sigma_{xy}+C_{2}\right)}{\left(\mu_{x}^{2}+\mu_{y}^{2}+C_{1}\right)\left(\sigma_{x}^{2}+\sigma_{y}^{2}+C_{2}\right)}$$

The SSIM is integrated in the final loss function defined as:

(13) $$L=L_{cGAN}\left(G,D\right)+\lambda L_{Robust}\left(G,\alpha \right)+\left(1-SSIM\right)$$

### 3.3. Dataset

**Figure 4:**
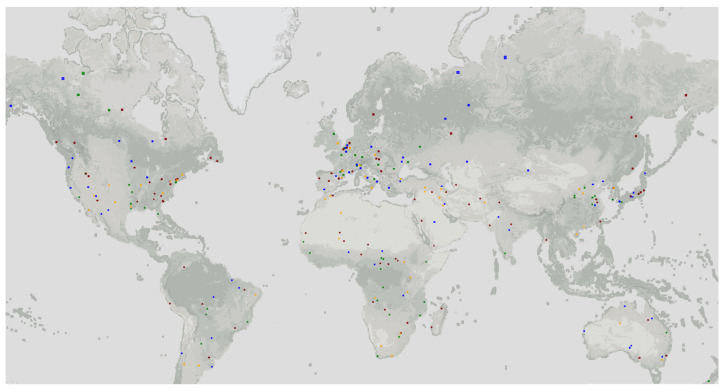
Visualization of the region of interest (ROIs) in the dataset. The visualization is realized in Google Earth Engine [52]. The colors represent different seasons: ■ Spring ■ Summer ■ Autumn ■ Winter.

To validate the method, we used the SEN12MS dataset, which is based on the Sentinel-1 and Sentinel-2 datasets [50]. The Sentinel-2 data from the SEN12MS dataset are level 1-C top of atmosphere (TOA) reflectance products. The TOA reflectance is the digital number (DN) divided by 10,000. This data format saves more memory compared with using the floating point format. It should be noted that this dataset is not atmospherically corrected. A cloud filter was applied during the data selection process to make sure the effect of clouds was minimized. The imagery has in total 13 bands with the spatial resolution ranging from 10 m to 60 m. In our experiment, we selected the red (R), green (G), blue (B) and near-infrared (NIR) bands with 10 m resolution for the experiment. Details of the band information can be found in Table 1. The dataset encompasses diverse areas, including desert, field, forests, urban areas and water bodies. The images in SEN12MS are distributed globally with varying longitudes and latitudes, and is categorised by seasons, as illustrated in Figure 4. The dataset is categorized into the four meteorological seasons of the northern hemisphere: winter (1 December 2016 to 28 February 2017), spring (1 March 2017 to 30 May 2017), summer (1 June 2017 to 31 August 2017), and fall (1 September 2017 to 30 November 2017).

**Table 1:** Data description from Sentinel-2 user handbook [51]. The wavelength is the center wavelength.

Bands	Wavelength (nm)		Bandwidth	Resolution
	S2A	S2B	(nm)	(m)
Red	664.6	664.9	31	10
Green	559.8	559.0	36	10
Blue	492.4	492.1	66	10
NIR	832.8	832.9	106	10

## 4. Experimental Section

In our previous work [17], we verified the plausibility of using a cGAN to generate an artificial NIR band, and achieved the best numeric results using the cGAN with a pixel discriminator and robust loss. However, only 300 images in summer were used for evaluation, which was insufficient for deep learning research. In this paper, we tested the method on the full autumn dataset (September 2017 to 30 November 2017) first, and then expanded the experiment to the full dataset (1 December to 30 November 2017).

### 4.1. One Season Experiment

To test the plausibility of the method, we first experimented with satellite images acquired in autumn to minimize the effect of land cover seasonal variance. Some land cover types, such as vegetation and water bodies, display different properties in different seasons. We selected all images acquired in autumn in the northern hemisphere for the training and testing. The images in the southern hemisphere were not selected because the season was the opposite. Among the training data, 10 percent of images were used for validation. The patch size was 256×256. The data fed into the network were normalized by the band-specific mean and standard deviation calculated in advance. The model was trained for 50 epochs with learning rate decay from the 25th epoch until it reached 0 in the last epoch. The λ in Equation (13) was set to 10 empirically. The initial learning rate was set to 0.002. The model was trained on a TITAN Xp GPU with 12 GB memory. In this experiment, we replaced all the batch normalization with instance normalization since the former produced unsatisfactory results, especially in the rule-based classification. We did not observe this issue in our previous work [17], possibly due to the small amount of test data that did not have large intra-variance.

### 4.2. Full Season Experiment

After experimenting with data from a single season, we moved further to using the full season data to test our method. Within the full season data, we could clearly see the distinct characteristics of the same land cover type in different seasons. We followed the same training protocol as in the one season experiment, except for training and testing with the full season data. The number of training images was 135,133 in total, among which 121,620 images were used for training and 13,513 for validation. The total number of test images was 45,529.

### 4.3. Evaluation Metrics

Various quantitative evaluation metrics were employed to assess the accuracy of our model. As is typical for GAN applications, our method aims to produce realistic-looking results. However, we did not assess the realism of the generated images through visual perception experiments, which are commonly used in other computer vision applications. In this subsection, we provide a detailed introduction to the evaluation metrics used in our study.

#### 4.3.1. Mean Absolute Error

Mean absolute error is a very common metric. It is intuitive and easy to calculate. It is defined as:

(14) $$MAE\left(x,y\right)=\frac{\sum_{i=1}^{n}\left|\hat{y_{i}}-y_{i}\right|}{n}$$

In Equation (14), $y_{i}$ is the true value and $\hat{y_{i}}$ is the predicted value. We use this metric to evaluate the quality of the generated images. The smaller MAE is, the more similar the generated image is to the original one numerically.

#### 4.3.2. Normalized Root Mean Squared Error

In addition to *MAE*, we also use *NRMSE*. It has many variants. In our evaluation, *NRMSE* is the root mean square error normalized by the difference between the minimum and the maximum of the theoretical NIR reflectance. In our case, the theoretical reflectance ranged from 0 to 1.

(15) $$NRMSE\left(x,y\right)=\frac{1}{y_{max}-y_{min}}\sqrt{\sum_{i=1}^{n}\frac{{\left(\hat{y_{i}}-y_{i}\right)}^{2}}{n}}$$

#### 4.3.3. Structural Similarity Index

The structural similarity index (SSIM) is used to evaluate the perceptual quality of simulated images based on the assumption that human visual perception is highly adapted for extracting structural information [49]. The definition is the same as for the SSIM loss function. See Section 3.2.3 for the detailed definition.

#### 4.3.4. Normalized Difference Vegetation Index (NDVI)

Different vegetation types demonstrate dissimilar responses to spectral signals, and phenology studies have shown that vegetation has a distinct response to seasonal variation. Therefore, vegetation indices are useful tools for analysing landcover types, especially vegetation. We evaluated the artificial *NIR* band using *NDVI* to test how well it could represent vegetation. It is calculated as:

(16) $$NDVI=\frac{NIR-Red}{NIR+Red}$$

#### 4.3.5. Normalized Difference Water Index (NDWI)

Another useful index based on spectral bands is *NDWI*. It has shown a significant capacity for highlighting water from remote sensing imagery. In the dataset, water bodies are a common landcover type. It can be calculated as:

(17) $$NDWI=\frac{Green-NIR}{Green+NIR}$$

#### 4.3.6. NDVI Based Classification

In addition to approximating the reflectance values, we expected the near-infrared channel to accurately reflect the characteristics of different land cover types. Specifically, the generated *NDVI* should have lower values for water bodies and higher values for vegetation areas. To evaluate this, we implemented a simple rule-based classification scheme that quantizes the *NDVI* and derives four classes: water, barren land, low vegetation, and high vegetation. The class definitions are shown in Equation (18). It is important to note that this classification scheme overgeneralizes all land cover types. However, since our goal was to assess whether the artificial *NIR* can distinguish classes with distinctive spectral characteristics, this classification scheme still provides a meaningful evaluation method. The four classes are derived according to the following rule:

(18) $$\begin{array}{cc} Pixel=Water,\; & if:\\ -1\leq & NDVI<-0.1\\ Pixel=Barren,\; & if:\\ -0.1\leq & NDVI<0.1\\ Pixel=LowVegetation,\; & if:\\ 0.1\leq & NDVI<0.4\\ Pixel=HighVegetation,\; & if:\\ 0.4\leq & NDVI\leq 1.0 \end{array}$$

We evaluated this 4-class classification result using the Jaccard index, also know as the intersection over union (IoU) score, which is widely used in semantic segmentation tasks. It is defined as:

(19) $$J=\frac{\left|x\cap y\right|}{\left|x\cup y\right|}$$

The Jaccard Index is the area of intersection between prediction (*x*) and ground truth (*y*) divided by the area of union of prediction and ground truth. It provides a fair evaluation even when the class distributions are unbalanced.

## 5. Results and Discussion

### 5.1. Result

#### 5.1.1. Results of Single Season Experiment

**Figure 5:**
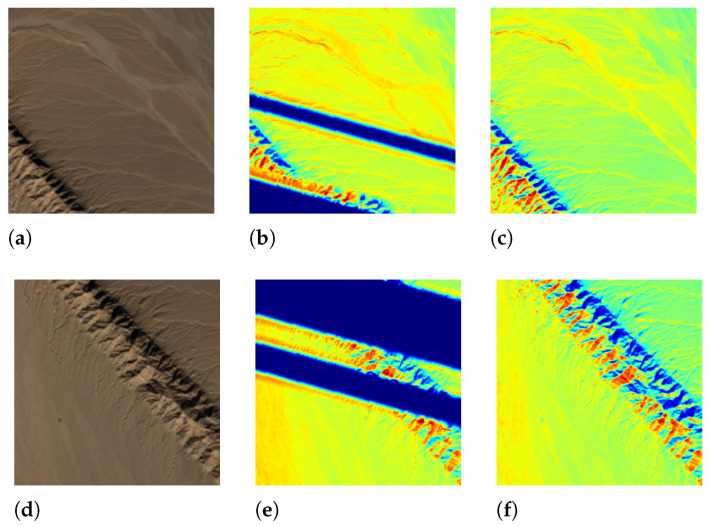
Selected example patches from Sentinel-2 dataset (**a**,**d**). Artifacts can be seen in the real *NIR* band (**b**,**e**). The corrupted pixels in the *NIR* band are recovered by the model (**c**,**f**).

**Figure 6:**
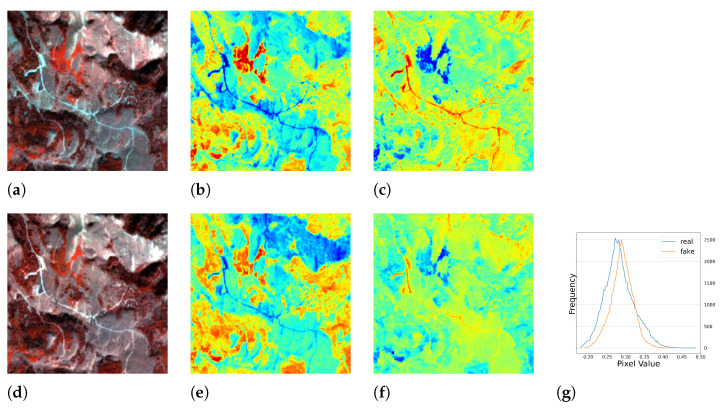
The single season result of cGAN-PixelD with robust loss. The ROI is located in Idaho, USA. (**a**) pseudo color image with the real *NIR* band; (**b**) *NDVI* from the real *NIR* band; (**c**) *NDWI* from the real *NIR* band; (**d**) pseudo color image with the fake *NIR* band; (**e**) *NDVI* from the fake *NIR* band; (**f**) *NDWI* from the fake *NIR* band; (**g**) histogram plot of the real and fake *NIR* bands.

**Figure 7:**
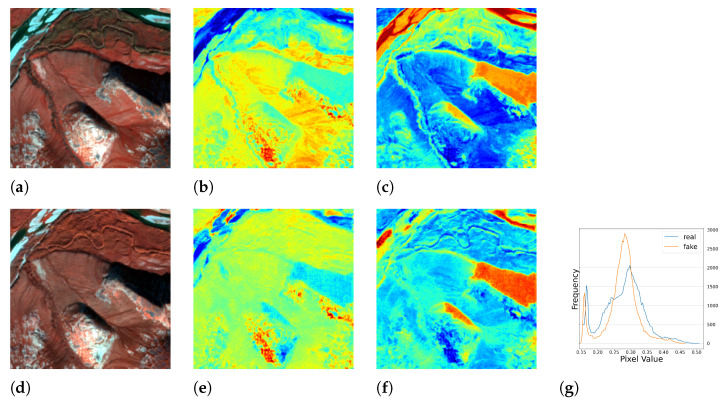
The single season result of cGAN-PixelD with robust loss. The ROI is located in the northeast of Russia. (**a**) pseudo color image with the real *NIR* band; (**b**) *NDVI* from the real *NIR* band; (**c**) *NDWI* from the real *NIR* band; (**d**) pseudo color image with the fake *NIR* band; (**e**) *NDVI* from the fake *NIR* band; (**f**) *NDWI* from the fake *NIR* band; (**g**) histogram plot of the real and fake *NIR* bands.

**Figure 8:**
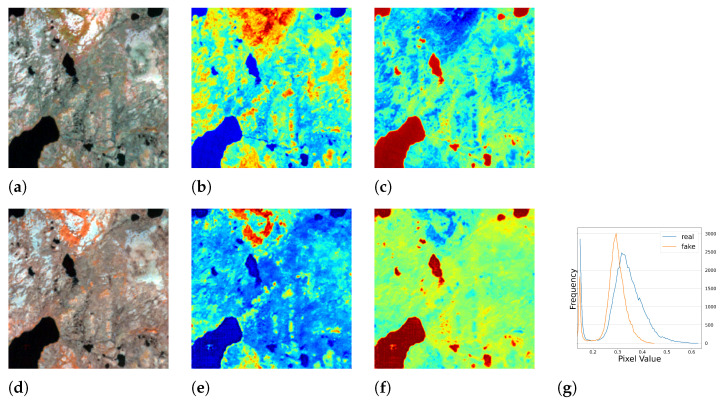
The single season result of cGAN-PixelD with robust loss. The ROI is located in Newfoundland and Labrador, Canada. (**a**) pseudo color image with the real *NIR* band; (**b**) *NDVI* from the real *NIR* band; (**c**) *NDWI* from the real *NIR* band; (**d**) pseudo color image with the fake *NIR* band; (**e**) *NDVI* from the fake *NIR* band; (**f**) *NDWI* from the fake *NIR* band; (**g**) histogram plot of the real and fake *NIR* bands.

**Figure 9:**
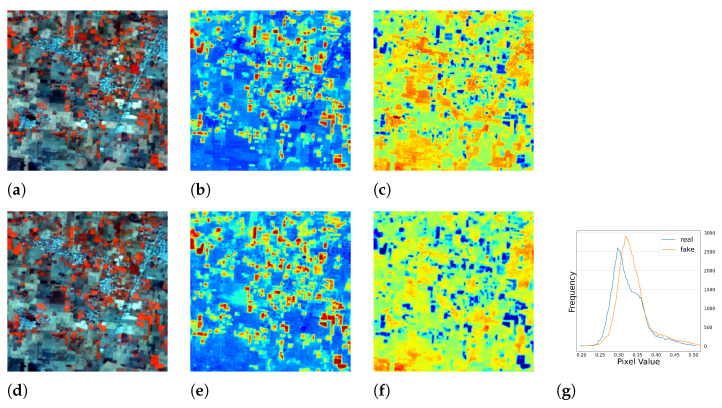
The single season result of cGAN-PixelD with robust loss. The ROI is located in Sialkot, Pakistan. (**a**) pseudo color image with the real *NIR* band; (**b**) *NDVI* from the real *NIR* band; (**c**) *NDWI* from the real *NIR* band; (**d**) pseudo color image with the fake *NIR* band; (**e**) *NDVI* from the fake *NIR* band; (**f**) *NDWI* from the fake *NIR* band; (**g**) histogram plot of the real and fake *NIR* bands.

Surprisingly, a serendipitous outcome of the method arose during the analysis of the images with the worst performance. As shown in Figure 5, the test images had blank values that were probably caused by errors in the data down-link or data processing. With the complete RGB bands, the model was able to recover the corrupted *NIR* band. This discovery suggested another use case for the method. Representative image patches are presented in Figure 6, Figure 7, Figure 8 and Figure 9, which are located in the United States, Russia, Canada, and Pakistan. The images are visualized in pseudo-color and the Jet color map is used for indices visualization. In general, the results appear realistic without distinguishable fake traits. As shown in Table 2, the cGAN-PixelD model with GAN loss, robust loss, and structural loss outperformed the other model variants. In Figure 6, Figure 7, Figure 8 and Figure 9, the generated *NIR* bands can highlight ground objects that are salient in *NDVI* and *NDWI*. However, some inaccuracies are also observed. For example, in Figure 7f, only a portion of the river is highlighted. Histograms of the real and artificial *NIR* bands show that, while the fake *NIR* band can capture the overall distribution of the data, there are still significant shifts from the real distribution in some intervals.

#### 5.1.2. Results of Multi-Season Experiment

**Figure 10:**
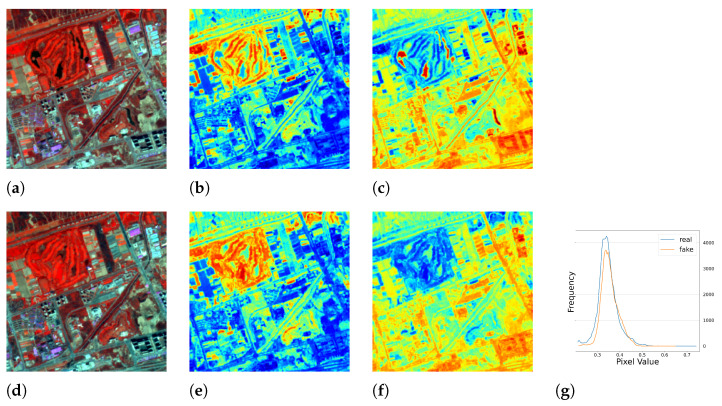
The full season result of cGAN-PixelD with robust loss, spring. The ROI is located in Xi’an, China. (**a**) pseudo color image with the real *NIR* band; (**b**) *NDVI* from the real *NIR* band; (**c**) *NDWI* from the real *NIR* band; (**d**) pseudo color image with the fake *NIR* band; (**e**) *NDVI* from the fake *NIR* band; (**f**) *NDWI* from the fake *NIR* band; (**g**) histogram plot of the real and fake *NIR* bands.

**Figure 11:**
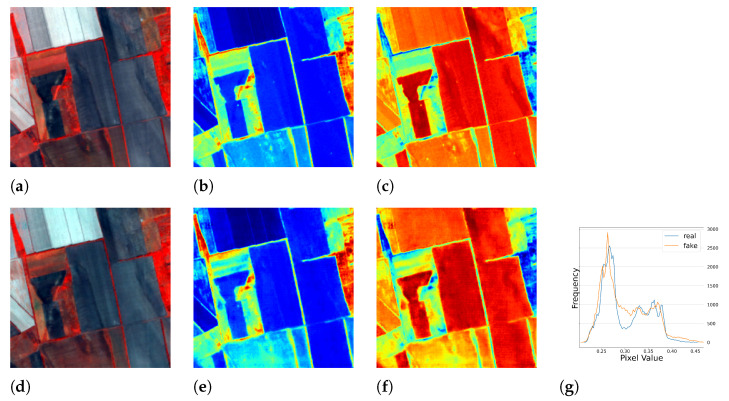
The full season result of cGAN-PixelD with robust loss, summer. The ROI is located in Novosavyts’ke, Ukraine, near the border with Moldova. (**a**) pseudo color image with the real *NIR* band; (**b**) *NDVI* from the real *NIR* band; (**c**) *NDWI* from the real *NIR* band; (**d**) pseudo color image with the fake *NIR* band; (**e**) *NDVI* from the fake *NIR* band; (**f**) *NDWI* from the fake *NIR* band; (**g**) histogram plot of the real and fake *NIR* bands.

**Figure 12:**
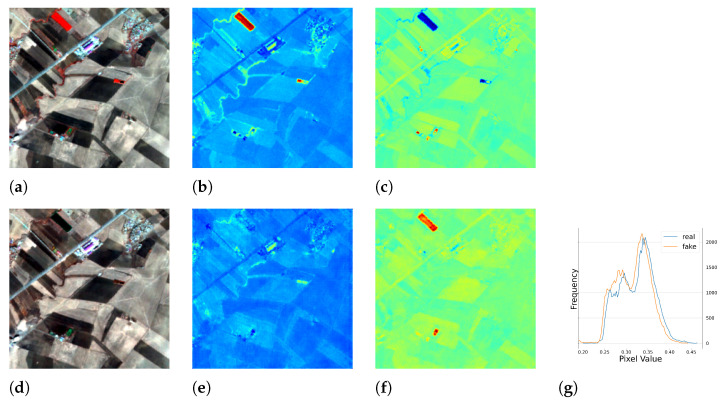
The full season result of cGAN-PixelD with robust loss, autumn. The ROI is located in Qararnaz, Iraq. (**a**) pseudo color image with the real *NIR* band; (**b**) *NDVI* from the real *NIR* band; (**c**) *NDWI* from the real *NIR* band; (**d**) pseudo color image with the fake *NIR* band; (**e**) *NDVI* from the fake *NIR* band; (**f**) *NDWI* from the fake *NIR* band; (**g**) histogram plot of the real and fake *NIR* bands.

**Figure 13:**
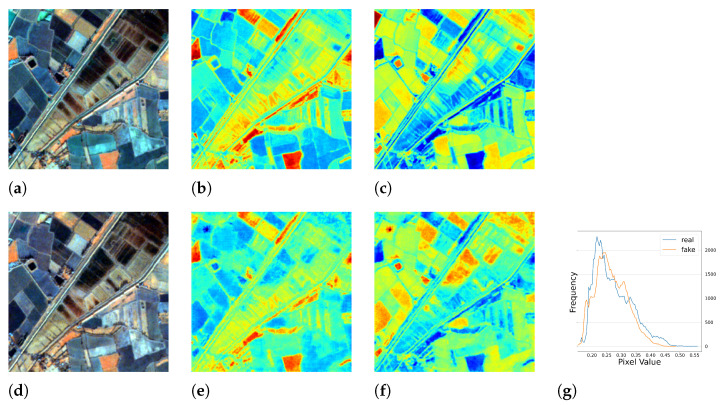
The full season result of cGAN-PixelD with robust loss, winter. The ROI is located in East Cambridgeshire, the United Kingdom. (**a**) pseudo color image with the real *NIR* band; (**b**) *NDVI* from the real *NIR* band; (**c**) *NDWI* from the real *NIR* band; (**d**) pseudo color image with the fake *NIR* band; (**e**) *NDVI* from the fake *NIR* band; (**f**) *NDWI* from the fake *NIR* band; (**g**) histogram plot of the real and fake *NIR* bands.

We conducted further experiments using full season data to test the performance of our model under seasonal influence. The results are presented in Table 2 and Figure 10, Figure 11, Figure 12 and Figure 13, which demonstrate that the model was able to produce reasonable results despite the varying responses of ground objects to seasonal changes. Notably, using the full season dataset achieved better quantitative results than using only autumn data. However, in some cases, the model failed to accurately capture the characteristics of the ground objects. For example, as shown in Figure 10c, small water bodies were not correctly reflected in the generated *NDWI*. After inspecting the satellite image of this area, we discovered that these water bodies had a similar color to vegetation due to high pollution levels, the presence of algae or aquatic plants, leading to the model’s failure to generalize and produce an *NIR* response similar to vegetation. Another error can be observed in Figure 12c,f, where the real and fake *NDWI* contradict each other. Upon visual inspection of the region of interest using Google Earth, we could not identify the object but can only assume that it was a man-made object that strongly reflected the *NIR* band. Similar to the single season experiment, the generated *NIR* band generally approximated the overall distribution of the real data. However, in some intervals, the shift from the real distribution was still noticeable.

#### 5.1.3. Results of Different Satellite Experiment

We also conducted tests to evaluate the generalization ability of our model trained with the Sentinel-2 dataset on the Landsat 8 dataset. As we expected, the model failed to generalize well to the Landsat 8 dataset. This difference could be attributed to various factors, such as differences in illumination, atmospheric or sensor conditions. For instance, Sentinel-2 and Landsat 8 OLI have slightly different bandpasses, and different measuring times can result in different solar angles. Additionally, the ground sampling distance of Landsat 8 is 30 m for corresponding bands, which can cause mixed pixels to have a different spectral response than pure ones.

### 5.2. Reflections on Deficiency of the Method

From the previous section, we can see that the proposed method can work reasonably well for the same sensor. The numeric evaluation demonstrated the effectiveness of the method and its potential to some degree. The model is an end-to-end trainable black box, which is difficult to interpret. However, instead of reiterating the effectiveness of the method, we want to focus more on the limitations. We hereby address some aspects that need special consideration in future work.

#### 5.2.1. Discussion on Atmospheric Correction

In remote sensing datasets, ground objects often exhibit vastly different responses to the sensor when atmospheric effects are present, adding to the complexity of the problem. In our experiment, we utilized the non-atmospheric corrected dataset for several reasons. Firstly, the primary objective of this research was to test the feasibility of utilizing GANs to simulate the *NIR* signal received by the sensor, irrespective of whether it was surface reflectance or radiance. Therefore, atmospheric correction was not required in this problem setting. Secondly, the *NIR* band we aimed to synthesize had a wavelength of approximately 800 nm, and its response to atmospheric effects was lower than that of the RGB bands. Even if the atmospheric effects were substantial in some scenes, the signal received by the sensor for the same ground object would not differ significantly. Furthermore, we employed a dataset that excluded images with severe haze or cloud effects, minimizing the influence of the atmosphere on the signal received by the sensor. Lastly, atmospheric correction techniques are not always 100 percent accurate and require various parameters, thereby complicating the problem. In some remote sensing applications, atmospheric correction is not essential. For instance, it has been argued that atmospheric correction does not improve land cover classification accuracy [53]. In multi-temporal change detection, atmospheric correction is typically unnecessary. When the training and testing data originate from different locations and times, a simple dark subtraction method is usually sufficient [54]. However, we still believe that using atmospherically corrected data could enhance the model’s performance. Atmospheric correction removes the effects of atmosphere on the signal received by the sensor. Atmospheric effects are caused by water drops, aerosols, and gas molecules, which could scatter, refract or absorb the sunlight. When such effects are removed, the problem of simulating spectral bands simply boils down to injecting spectral features into each land cover type, despite the difficulty in classifying numerous ground objects accurately. We expect less error with atmospherically corrected data. However, this statement remains to be substantiated with further investigation. The biggest constraint is the fact that not every band can be inferred from other bands, as observed in the case of water. For example, short-wave near-infrared (SWIR) bands can pass through haze and fire smoke while visible bands can not. In this scenario, the SWIR band cannot be generated from the RGB bands accurately.

#### 5.2.2. Discussion on Transferring to a Different Dataset

We tested the model trained on Sentinel-2 data on the Landsat-8 dataset, but it did not perform well due to the distinct characteristics of the two types of imagery. This problem cannot be solved without integrating professional knowledge in sensor and signal processing. However, a harmonization workflow has been proposed to enable the simultaneous use of Landsat and Sentinel-2 for land cover monitoring [1]. This workflow involves radiometric and geometric adjustments to provide a consistent surface reflectance record for time-series applications. First, the data is cloud-masked and atmospherically corrected using a radiative transfer algorithm. Then, the data is normalized to a common nadir view geometry using bi-directional reflectance distribution function (BRDF) estimation. Finally, the spectral bandpass adjustment is applied to Landsat-8 Operational Land Imager (OLI), and both Sentinel-2 and Landsat-8 datasets are gridded to a common resolution, projection, and spatial extent. This workflow sheds light on using a model trained on different data for *NIR* band generation, and it could be integrated into the design of sensors that do not capture *NIR* bands. In the future, it would be worthwhile to investigate the performance of this model in the context of drone imagery. If applicable, this approach could greatly expand the applications of old data.

## 6. Conclusions

In this paper, we propose an innovative application of conditional generative adversarial network in multispectral remote sensing. We investigate the plausibility of simulating Sentinel-2 *NIR* band TOA reflectance from RGB bands using a cGAN. Furthermore, we replace the traditional loss function with a robust loss function and a structural similarity loss function to further improve the results. We also extend the scale of the experiment data and the scope of analysis compared with our pilot study, which was intended to examine the plausibility of this application.

We tested the proposed framework on SEN12MS multispectral datasets and achieved reasonable results, demonstrating the potential of applying this framework in various tasks, such as supplementing low-cost sensors with additional bands, recovering corrupted band information, and deriving supplemental indices for landcover classification. Although the method demonstrated reasonable results, we believe that additional knowledge is essential for more accurate results. Specifically, knowledge in the fields of signal processing, sensor calibration, and atmospheric modeling could contribute to a more robust model. In the meantime, the limitations of the method should be highlighted. In space-borne imaging, atmospheric effects play a crucial role in characterizing ground objects. These effects can result in distinct representations of ground objects by the same spectral bands, leading to wrong outputs. More importantly, the black box model might be able to imitate the human visual system, but whether it can accurately model physical phenomenon remains to be answered. Nevertheless, this framework could have greater potential in airborne datasets where the atmospheric effect is not as significant as on a spaceborne sensor. In addition, the possibility of combining ground object measurement and imagery captured by a spaceborne sensor can help to calibrate and fine-tune the model for higher accuracy. It could be applied to supplement airborne datasets for better characterisation of ground objects. In the future, we will seek to test the method on a multispectral airborne dataset to derive a plausible method to supplement a visible camera with additional artificial spectral bands to expand the usage of history data. We will also further investigate the performance of the generated *NIR* band in large-scale vegetation monitoring and supervised landcover classification.

## Figures and Tables

**Table 2:** The results of autumn and full season. G, R, S denote GAN loss, robust loss and structural similarity loss, respectively.

		MAE	MAE	MAE	NMSE	SSIM	IoU
		($\times 10^{-3}$)	($\times 10^{-3}$)	($\times 10^{-3}$)	(%)	(%)	(%)
		*NIR*	*NDVI*	*NDWI*	*NIR*		
**Network**	**Loss**						
**Autumn**							
cGAN-PixelD	G+R	22.91	32.01	33.90	2.88	90.88	84.41
	G+R+S	22.92	31.70	33.56	2.88	90.95	84.37
cGAN-PatchD	G+R	25.46	35.24	37.31	3.19	87.90	82.71
	G+R+S	25.02	34.48	36.48	3.14	88.30	83.12
**Full Season**							
cGAN-PixelD	G+R+S	23.78	28.06	30.40	3.00	89.98	89.50

## Data Availability

The SEN12MS dataset can be downloaded at https://github.com/schmitt-muc/SEN12MS, accessed on 10 April 2023.

## Abbreviations

The following abbreviations are used in this manuscript:

SLR	Single-lens reflex
GAN	Generative adversarial network
cGAN	Conditional generative adversarial network
DSM	Digital surface model
TOA	Top of atmosphere
*NIR*	Near-infrared
RGB	Red, green and blue
*NDVI*	Normalized difference vegetation index
*NDWI*	Normalized difference water index
